# Synthesis of active packaging films from *Lepidium sativum* gum/polyvinyl alcohol composites and their application in preserving cheddar cheese

**DOI:** 10.1038/s41598-023-28173-3

**Published:** 2023-01-30

**Authors:** Mona Abdel Rehim, Hamdy A. Zahran, Marwa Al-Moghazy

**Affiliations:** 1 Packing and Packaging Materials Department, National Research Centre, Dokki, 12622 Cairo Egypt; 2 Fats and Oils Department, Food Industries and Nutrition Research Institute, National Research Centre, Dokki, 12622 Cairo Egypt; 3 Dairy Department, Food Industries and Nutrition Research Institute, National Research Centre, Dokki, 12622 Cairo Egypt

**Keywords:** Biochemistry, Microbiology, Biochemistry, Green chemistry, Materials chemistry

## Abstract

The interest in active packaging for extending food shelf life has increased lately. Moreover, the negative impact of synthetic plastic wastes on the environmental motivated the researchers to seek for bio-based alternatives. In this context, active packaging film made of a composite composed of *Lepidium sativum* extract (LSE), polyvinyl alcohol (PVA), and a fixed amount of hyperbranched polyamide amine (PAMAM) were prepared. The chemical, thermal, and mechanical properties of the film were investigated. Moreover, we examined the extract’s constituents and antioxidant properties. Cheddar cheese samples were coated with films of different compositions. The samples coated with active packaging films showed a longer preservation time of up to 4 weeks compared to other samples, which noticeably deteriorated. The films showed potent antimicrobial activity against five food-borne bacteria: three gram-negative bacteria including *Escherichia coli* O157.H7, *Pseudomonas aeruginosa*, and *Salmonella* Typhimurium, and two gram-positive bacteria, *Listeria monocytogenes,* and *Staphylococcus aureus*. Applying PVA films containing LSE improved the microbiological quality and delayed the visible decay of cheddar cheese. The oxidizability of the fat extracted from different cheese samples was 0.40–0.98, confirming oxidation resistance. Finally, cheese samples coated with treated films were protected from forming *trans* fats compared to other samples, demonstrating the effectiveness of modified films as antioxidant, antimicrobial, and food-preserving packaging.

## Introduction

Packaging materials based on bio-resources are of increasing interest as sustainable alternatives to petroleum-based polymers that yield enormous amounts of plastic waste. Efforts have been made to develop biodegradable packaging materials with properties comparable to those of synthetic materials^1,2^. Researchers and food industry packaging producers are investigating abundant, low-cost, biodegradable alternatives to plastic packaging to reduce CO_2_ emissions^3^. Plants contain biologically active beneficial compounds used in the pharmaceutical, food, or cosmetic industries. These compounds include phenols and flavonoids that have antimicrobial and antioxidant properties. They can be used in food packaging as an edible coating^3^ or as packaging film to prolong food shelf life and prevent spoiling^5^. *Lepidium sativum*, or cress seed, is a Cruciferae family member that grows in Egypt, Europe, and other parts of the world^4^. The seeds have been used in traditional medicine to treat hepatotoxicity, asthma, fractures, hyperglycemia, and enuresis^7,8^. *L. sativum* gum can be extracted as *L. sativum* extract (LSE) using solvents like water, ethanol, and supercritical CO_2_. Fractionation results of the extracted hydrocolloids demonstrated that the fractions had different physicochemical and functional properties^5–8^. Additionally, an investigation of sugar composition, uronic acid content, functional groups, and molecular weights has been carried out^8^. Stepwise fractionation using water illustrated that the chemical composition, including monosaccharides, moisture, ash, nitrogen, carbon, and uronic acid levels, of the fractions varied significantly. The potential applications of cress seed gum fractions are in food emulsions and foam systems as a thickener and stabilizer.

Cheese is a popular ready-to-eat food consumed without heat treatment, and its nutritional value depends mainly on milk characteristics and production technology. Essential nutrients, including proteins, bioactive peptides, amino acids, fat, fatty acids (FAs), vitamins, and minerals, are found in cheese^9^. Milk fat is the most complex fat in the human diet, as it contains > 400 distinct FAs^10^. However, saturated FAs (SFAs) are the predominant class of FAs in milk fat and include short-chain FAs (SCFA), medium and long-chain FAs, as well as odd FAs^10,11^. The best source of natural *trans* FAs, such as vaccenic acid (trans11 C18.1) and conjugated linoleic acid (cis9 trans11 C18.2), is milk fat, and these exhibit favorable properties compared to artificial *trans* FAs in partially hydrogenated oils^12^.

Currently, the most important pathogens in the food industry are *Salmonella*, *Listeria monocytogenes*, *Staphylococcus aureus*, and *Escherichia coli* O157.H7. These pathogens are known to be associated with food-borne disease outbreaks that cause illness in about 600 million people worldwide yearly^13^. Cheese can be a carrier for food-borne bacteria such as Shiga toxin-producing *E. coli*, *L. monocytogenes*, *Salmonella spp*., and *S. aureus*^14^. Mold development may pose a health concern due to the probability of mycotoxin secreted by some mold species^15^. Cross-contamination with food-borne bacteria can occur in cheese during handling and storage. Visible cheese spoilage usually starts on superficial parts of the cheese^16^; thus, protecting the cheese surface from microbial contamination is an effective strategy to eliminate cross-contamination and spoilage^17^.

Antimicrobial active packaging enhances microbiological quality, prolongs the shelf life, and limits pathogenic bacteria cross-contamination. Usually, these active films are designed by incorporating antimicrobials, such as organic acids, enzymes, bacteriocins, and natural extracts, into packaging materials to control the release of antimicrobial agents to the food surface^18^. Karamkhani et al.^19^ described the incorporation of *L. sativum* in an edible film containing different ratios of carvacrol. The edible coating was used to protect and enhance the shelf life of refrigerated farmed shrimp. The films showed antimicrobial and antioxidant properties enhanced as the ratio of carvacrol increased.

This study investigated the antioxidant and antimicrobial properties of packaging films composed of polyvinyl alcohol (PVA), LSE, and a fixed amount of hyperbranched polyamide amine (PAMAM) as a plasticizer. The chemical, mechanical, and thermal properties of the prepared films were studied. The films were used to preserve cheddar cheese. After packaging, the biological properties of the films and the cheese quality were investigated.

## Experimental methods

### Materials

*L. sativum* seeds were cultivated in Egypt and purchased from local market in Cairo. The seeds were authenticated at Herbarium of Plant Chemistry and Systematics Department, National Research Center (NRC), Egypt. PVA (degree of polymerization = 1700–1800) was obtained from Qualikms Fine Chemicals Pvt. Ltd., New Delhi, India. Hyperbranched PAMAM was prepared and characterized in-house according to a reference^20^. All solvents were used as received. Five food-borne bacterial species were used in this study, including three gram-negative strains, *E. coli* O157.H7 strain 93,111, *P. aeruginosa* ATCC 9027, and *S.* Typhimurium ATCC 14,280, and two gram-positive strains, *L. monocytogenes* ATCC 7644 and *S. aureus* ATCC 25,923. All strains were stored at − 20 °C in a tryptone soy broth medium containing 15% glycerol. The Microbiology Department, Faculty of Agriculture, Cairo University, generously provided all bacterial strains.

### Methods

#### Preparation of packaging films from LSE and PVA

LS seeds (5 g) were soaked in water and incubated overnight. The extract was separated by filtration and used to prepare the casting solutions. An appropriate amount of PVA was dissolved in hot water, a definite volume of the PVA solution was mixed with LSE according to the ratio presented in Table 1. It was stirred thoroughly with a magnetic stirrer. 0.5 g PAMAM was added as a plasticizer, and 0.5 g citric acid was added for cross-linking. The solution was mixed well. Then, air bubbles were removed with a vacuum. The solution was poured into glass dishes and left to dry at room temperature and then in an oven at 60 °C.

**Table 1:** Analysis of phenolic compounds of LSE using HPLC.

Compound	Retention time Rt (min)	Area%	Concentration (µg/g)
Protocatechuic acid	6.6	136.6	149.7
p-hydroxybenzoic acid	9.8	725.4	698.6
Catechin	11.7	70.3	186.8
Chlorogenic acid	12.4	87.5	63.9
Caffeic acid	13	139.9	50.1
Syringic acid	14.4	7.2	4.5
Vanillic acid	15.6	42.7	17.1
Ferulic acid	20.2	85.7	30.3
Sinapic acid	20.9	3376.7	1310.1
p-coumaric acid	24.2	35.8	28.3
Rutin	23.1	6065.6	7525.6
Cinnamic acid	34.5	87.9	38.3

#### Antioxidant activity of the films

The antioxidant capacity of prepared active films was measured according to a method described elsewhere^21^. The freshly prepared 2,2 diphenyl-1-picrylhydrazyl (DPPH) solution (6 × 10^−5^ M in methanol) was vortexed and left at ambient temperature for 30 min in the dark. The film samples (about 0.2 g) were weighed in a glass tube (30 mL) and added to a 4 mL DPPH solution (diluted). The percent of DPPH inhibition was determined by measuring the absorbance of the samples at 517 nm. The control sample (without film) was measured as well. The inhibition percent of the DPPH radicals was calculated using the following equation:

$${\text{DPPH Inhibition }}\% \, = \, \left[ {\left( {{\text{A}}_{{{\text{Control}}}} {-}{\text{A}}_{{{\text{Sample}}}} } \right)/{\text{ A}}_{{{\text{Control}}}} } \right] \, \times { 1}00,$$

where A is the absorbance.

#### Antimicrobial activity of the prepared films

The antimicrobial properties of the films were evaluated according to^22^. The films were sterilized under UV light for 30 min on each side, and 0.15 g of each film was inserted into a 24-well microplate. 2 mL nutrient broth was added to each well, then inoculated with 20 µL bacterial culture (incubated for 24 h at 37 °C), and diluted to a final concentration of ≈10^6^ CFU/mL, determined by counting. The test was performed in triplicate. After incubation for 24 h at 37 °C, the bacterial count of each sample was determined using the drop plate method^23^.

#### Application to cheese

Cheddar cheese was cut into equal square slices of 4 × 4 cm, and films of the same dimensions were fixed on the upper and lower sides. These were kept in 60-mm disposable Petri dishes, sealed with parafilm, and stored in a refrigerator at 4 °C. The cheese samples were divided into three groups: (C) without film (B) covered with film and (T) covered with film A1. Microbiological analysis was performed weekly following total mesophilic, psychrotrophic yeast, mold, and coliform counts. Different cheese samples were minced aseptically, and then 10 g of each were transferred into 90 mL sterilized trisodium citrate solutions (2% w/v) and homogenized by a stomacher for 1 min. Decimal dilutions were made with sterilized saline. Total mesophilic and psychrotrophic bacteria were enumerated on a standard plate count agar (Aldrich) after incubation at 30 °C for 48 h and 7 days at 4 °C, respectively. Potato dextrose agar (Conda Spain) acidified with 1% of 10% lactic acid solution (w/v) was used to count yeast and mold after incubation at 25 °C for 5 days. The coliform count was determined using a double layer of violet-red bile agar incubated at 37 °C for 24 h.

#### Decay evaluation

Fifteen cheese samples in each treatment group were observed to detect visible microbial growth. The number of spoiled cheese samples was counted, and the spoilage percentage was calculated by dividing the number of spoiled samples by the total number of cheese samples in each treatment group^24^.

#### Statistical analysis

The experimental data were evaluated using analysis of variance (ANOVA) and the Duncan test, and CoSTAT software was used to detect significant differences among means from three replicates at p < 0.05.

#### Lipid extraction

The Folch technique^25^ was used to extract fat from the evaluated cheeses. Each sample was ground homogenously. Approximately 3 g of the material was transferred into a 100-mL beaker and homogenized for 1 min with 30 mL methanol (IKA Ultra-Turrax^®^T18 digital). Following that, 30 mL chloroform was added, and the operation was repeated for another 2 min. A 250-mL glass beaker was used to filter the produced liquid. The solid residue was homogenized for 3 min in a 60 mL chloroform:methanol (2:1 v/v) mixture. The mixture was poured into a single cylinder. The complete filtrate was added to a solution of 0.88% NaCl_2_ in water (determining 14 volumes of filtrate), agitated, and left overnight. The upper layer was removed, and a combination of water and methanol (1:1 v/v) was added to the lower layer before repeated washing. The residual layer was filtered with anhydrous sodium sulfate over filter paper, and the solvent was then distilled.

#### Calculated oxidizability (Cox) value

The Cox value of the oils was calculated from the fatty acid percent according to Fatemi and Hammond^26^ using the following equation:

$${\text{Cox value }} = \, \left[ {\left( {{1 } \times {\text{ C18}}.{1}} \right) \, + \, \left( {{1}0.{3 } \times {\text{ C18}}.{2}} \right) \, + \, \left( {{21}.{6 } \times {\text{ C18}}.{3}} \right)} \right]/{1}00$$

### Equipment

The chemical structure of the prepared materials was studied with a Bruker VERTEX 80 ATR-FTIR instrument (Germany) combined with Platinum Diamond ATR, comprising a diamond disk. The internal reflector has a range of 400–4000 cm^−1^ with a resolution of 4 cm^−1^ and a refractive index of 2.4. Thermal stability was investigated using a thermogravimetric analyzer (TGA), with a heating range from room temperature to 700 °C and a heating rate of 10 °C/min under an N_2_ atmosphere. The mechanical properties of the films were measured with an INSTRON mechanical tester, five samples were measured for each film. UV analysis was measured using a spectrophotometer (Shimadzu, Germany). The extract fingerprint was investigated using HPLC analysis using an Agilent 1260 series. The separation was carried out using an Eclipse C18 column (4.6 mm × 250 mm i.d., 5 μm). The mobile phase consisted of water (A) and 0.05% trifluoroacetic acid in acetonitrile (B) at a flow rate of 1 ml/min. The mobile phase was programmed consecutively in a linear gradient as follows: 0 min (82% A); 0–5 min (80% A); 5–8 min (60% A); 8–12 min (60% A); 12–15 min (85% A), and 15–16 min (82% A). The multi-wavelength detector was monitored at 280 nm. The injection volume was 10 μl for each of the sample solutions. The column temperature was maintained at 35 °C.

The fatty acid composition was determined using the procedures described by Zahran and Tawfeuk^27^. The fatty acid methyl esters of the oil samples were analyzed for their constituents by GLC-FID (HP 6890 Gas Chromatography occupied with flame ionization detector, Hewlett Packard, USA). The injection volume was 1 μL with a splitting ratio of 50.1. A capillary column Supelco™ SP-2380 (60 m × 0.25 mm × 0.20 μm, Sigma-Aldrich, USA) was used, and the detector and injector temperatures were set at 250 °C. The thermal program was started at 100 °C and raised to 230 °C at a rate of 15 °C/min (hold for 30 min). The carrier gas was helium at a flow rate of 1.2 mL min^−1^.

### Human ethics and consent to participate

Not applicable for this study. Confirming all experimental research including the collection of plant material, complies with relevant institutional, national, and international guidelines and legislation.

## Results and discussion

### HPLC analysis of LSE

Aqueous LSE was extracted from the whole seed by distilled water at room temperature. The simple extraction procedure yielded natural compounds of antimicrobial activity and low cytotoxicity^28^. The composition of the obtained extract was analyzed using HPLC. Twelve phenolic compounds were detected in the seed extract (Table 2). Rutin, sinapic acid, and p-hydroxybenzoic acid were the most abundant phenolic compounds in the water extracts of cress seed, in agreement with^29^ Abdel-Aty et al. and Kaiyrkulova et al.^30^.

**Table 2:** Composition, thermal and mechanical characteristics of prepared films.

Sample	PVA solid content	Ratio of PVA:LS gum	10% wt. loss (°C)	Tensile strength (MPa)	Displacement at break (mm)
A	10%	1.0	306	53.2 ± 8.2^a^	60.7 ± 16.7^a^
A1	10%	1.1	287	27.9 ± 3.6^b^	30.4 ± 3.1^b^
A2	5%	1.1	295	23.3 ± 3.5^b^	29.9 ± 7.4^b^
A3	5%	1.2	189	Not measured	–

Results represent the means of 3 replicates ± sd.

Different superscript letters indicate significant differences (p < 0.05).

### Preparation of active packaging films

**Scheme 1:**
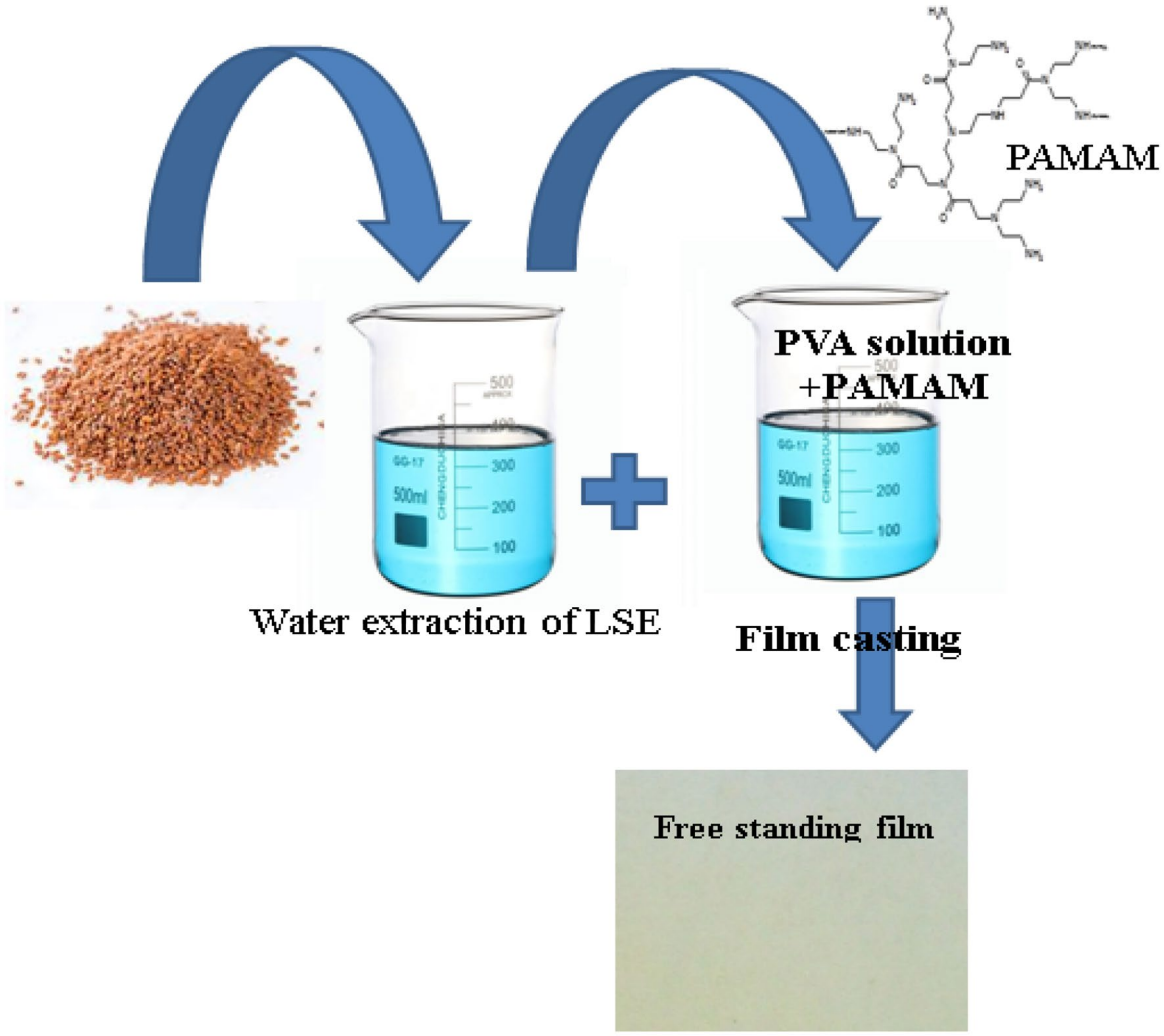
Preparation steps for achieving free-standing films suitable for food packaging.

Free-standing films from LSE/PVA blends were prepared according to Scheme 1. Although PVA acted as a suitable holding matrix for the extract, the optimal concentration of PVA needed to be identified to obtain a high-quality film. The first trials for film preparation were unsuccessful due to the high stiffness of the formed films. The concentrations of the PVA solutions and their ratio to the extract are shown in Table 2. The film F1 contained 10% PVA solution, and this amount was reduced to 5% in F2. The first film was much better than the second one. However, film F3 contained the same amount of PVA as F2 but double the amount of the extract, which worsened its appearance and mechanical properties. Hyperbranched polymers are a class of macromolecules characterized by their low solution and melt viscosity and the presence of many functional terminals^31^. Thus, hyperbranched PAMAM was added in small amounts to the film formulations for several reasons. It can act as a plasticizer and hence improve the mechanical properties of the film, it is not toxic^32^, and its amine end groups might have biological activity (its structure is shown in Scheme 1).

**Figure 1:**
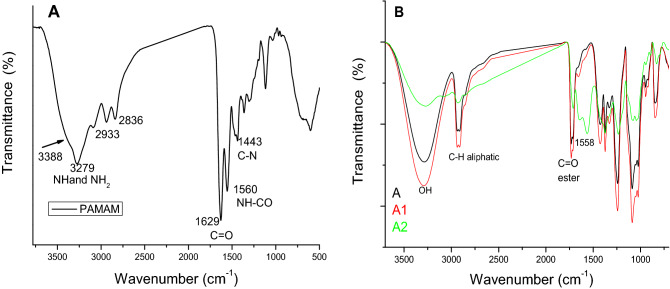
FTIR spectra of: (**A**) PAMAM and (**B**) prepared films.

Chemical structure of PAMAM is investigated using FTIR and the spectrum shown in Fig. 1A reaveled bands at 3388 and 3279 cm^−1^ corresponding to NH and NH_2_ groups respectively and bands for aliphatic CH at 2933 and 2836 cm^−1^. A band for amide C=O can be observed at 1629 cm^−1^ while the band of NH–CO appears at 1560 cm^−1^. The chemical structure of the prepared films was studied using FTIR, and the spectra are shown in Fig. 1B. The three investigated films comprised pure PVA (A), A1, and A2. The band at 3300 cm^−1^ is attributed to the OH group, and bands that appear at 1725 cm^−1^ are characteristic of C=O (an ester group) formed due to crosslinking reaction of citric acid and PVA^33^. Additionally, the spectrum of film A2 showed larger and overlapping bands at 1490–1620 cm^−1^ corresponding to C–O, C–OH, and C–C due to flavonids in the LSE^34,35^. These strongly appear since this formulation contains a larger amount of extract compared to the PVA matrix. The broadening in the bands can be attributed to the intermolecular and intramolecular interacions with the PVA chains with no chemical reaction^36^. It should be pointed out that the characteristic bands of PAMAM are all overlapped by the polymer and LSE bands due to small amount of used PAMAM in the films’ formulations.

### Thermal analysis

**Figure 2:**
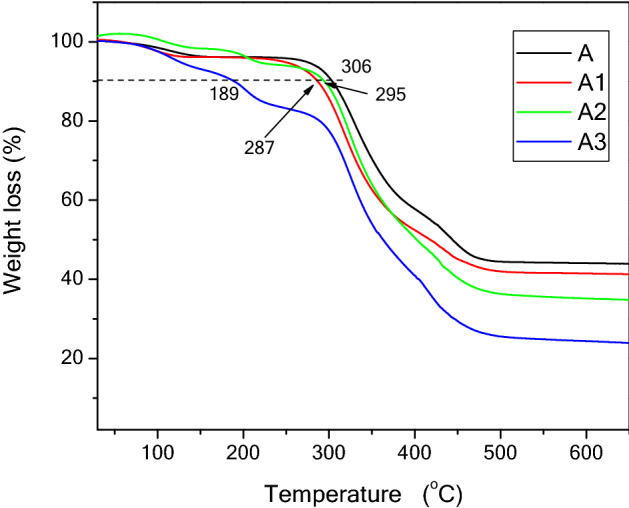
TGA of packaging films A1, A2, and A3 compared with pure film A.

The thermal stability of prepared films was studied using TGA, and the results are demonstrated in Fig. 2. Generally, all samples showed more than one degradation step and were thermally stable until nearly 300 °C, except film A3. The first degradation step around 120 °C can be attributed to water loss. However, an increasing amount of the extract in the film formulations decreased their thermal stability, as observed from the 10% weight loss (Table 2). The order of the film thermal stability was A > A1> A2 > A3. Nevertheless, film A3 showed early thermal degradation at 200 to 290 °C which can be attributed to degradation of LSE. It was previously reported that degradation of cress seed gum can occur in temperature above 200 °C due to disintegration of macromolecular polysaccharide chains (10). A last degradation step can be observed at 300 related to PVA chains.

### Mechanical testing and film morphology

#### Mechanical testing and film morphology

Studying the mechanical properties of a packaging film is essential to evaluate film performance. The mechanical properties represented in tensile strength (TS) and displacement percentage (D) of the composites were investigated. The results of TS analysis showed that increasing the amount of LSE in the film composition reduced the TS of the obtained film. Polyphenolic compounds can form hydrogen bonds with amino and hydroxyl groups in the polymer matrix or the plasticizer, weakening, the intermolecular interactions that stabilize the polymeric chains (Table 2). Moreover values obtained for D showed the same deceasing trend noting that, the difference in values between A1 and A2 is very small. This result can be attributed to presence of agglomrations that creat voids between the polymer matrix (PVA) and extract leading to easeier sample fracture^37^. Film A3 was composed of 75% LSE and 25% PVA, so it was thinner than the others and easy to rupture and consequently could not be measured by the mechanical tester.

**Figure 3:**
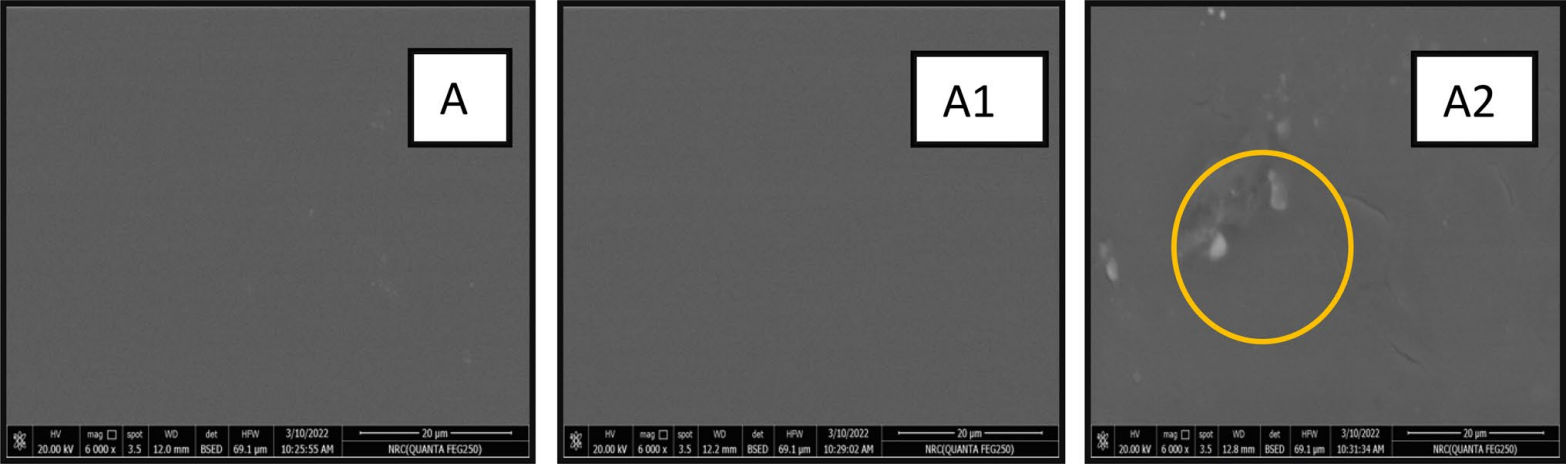
SEM images of samples A, A1, and A2 (*X* = 6000).

Investigation of the film surface morphology demonstrated that film A1 has a smooth and homogenous surface, unlike film A2, which revealed aggregates and small cracks (Fig. 3). The observed heterogeneous surface of A2 can be attributed to a lower amount of PVA, which works as a holding matrix for the extract. Moreover, structural changes in plant cells can occure in the air-dried films^38^. Hence, drying in air facilitates mass transfer through the solid matrix^28^.

### Antioxidant activity

**Figure 4:**
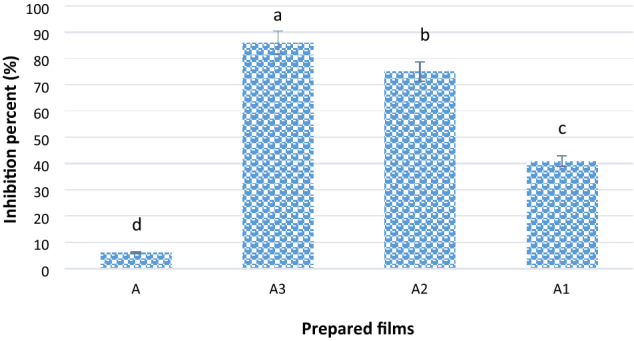
Inhibition percent of different prepared films.

Antioxidant activities of PVA/PAMAM composite films loaded with LSE at different ratios (A1, A2, and A3), as shown in Table 2, were evaluated using a DPPH free radical scavenging assay. The treated samples were compared with pure film A. The scavenging activity of DPPH (a stable free radical) in the presence of different films was determined by monitoring the decrease in its absorbance values at 517 nm. As shown in Fig. 4, the film (A3) exhibited higher antioxidant activity, followed by A2 and A1, compared to the pure film A. This can be attributed to the ability of LSE to donate active hydrogen atoms or transfer electrons to reduce the DPPH. The significantly higher antioxidant activity of the film A3 can also be attributed to the higher concentration of LSE in the film formulation.

### Antimicrobial activity of films

**Figure 5:**
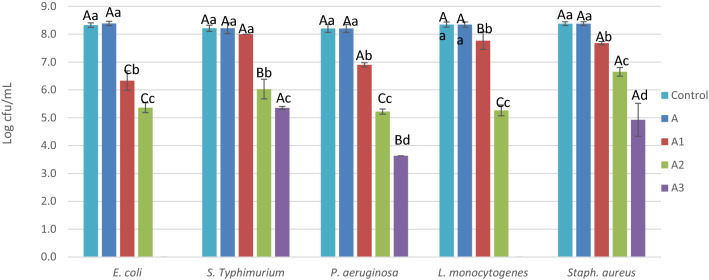
The antimicrobial effect of different films. Different capital letters indicate significant differences for bacterial counts within the same treatment. Small letters indicate significant differences for different treatments within the same bacteria (p < 0.05). Results represent the means of 3 replicates ± sd.

The antimicrobial activity of different films was tested against five food-borne bacteria (Fig. 5). PVA film A did not affect the bacteria counts compared to the control. The film A1 had antimicrobial activity against all tested bacteria except *S.* Typhimurium (p < 0.05). Additionally, A2 possessed antimicrobial activity against all tested bacteria. *E. coli* and *L. monocytogenes* were entirely suppressed by A3. The antimicrobial effect of different films containing different concentrations of LSE is ordered as follows: A3 > A2 > A1. The antimicrobial activity of LSE might be due to its high content of rutin, which has a high potent antimicrobial activity^39^. Abdelghany et al.^34^ found that incorporating LSE into PVA films increased their antimicrobial activity against *E. coli*, *S aureus*, and *P. aeruginosa*. Twelve phenolic compounds were detected in LSE (Table 2). Rutin, sinapic acid, and p-hydroxybenzoic acid, which are the most abundant phenolic compounds in the water extracts of *Lepidium* seed in agreement with^30,40^. Rutin was found to have antimicrobial activity aganst bacteria, yeast and mould^41^ The sinapic acid has been demonstrated antimicrobial activity in various studies on both plant and human pathogens^42^. P-hydroxybenzoic acid, a monohydroxy phenolic derivative of benzoic acid, is commonly used as antioxidant and antimicrobial in foods, beverages, medicines, and cosmetics^43^.

### Effect of PVA-LSE film on the microbial quality of cheese

According to the appropriate mechanical and physical properties of film A1, it was chosen as an active packaging film for cheese preservation. Evaluation of the effect of the PVA-LSE film on the microbial quality of the cheese was performed by counting the total mesophilic bacteria, psychrotrophic bacteria, yeast, mold, and coliform bacteria in the three groups of cheese, namely (C) control uncovered, (B) covered with PVA, and (T) covered with A1 (Table 3). After 2 weeks of cold storage at 4 °C, the C and B groups developed visible spoilage in all samples, so they were eliminated from the microbial analysis. For mesophilic bacteria, the counts increased significantly in all treatments with storage time. However, the T samples recorded fewer counts than groups C and B; moreover, T samples did not significantly increase from week 1 to 4 of cold storage (p < 0.05). The same observation was seen for psychrotrophic bacteria counts and the counts of yeast and mold. However, in the T treatment, the yeast and mold counts increased significantly until the 4th week of storage. Detecting coliform bacteria in cheeses and dairy products is a result of using raw milk or poor hygiene manufacturing and handling conditions^44^. Some states in the US have limits of 10 (1 log) or 100 (2 log) CFU/g for coliforms in cheese, while the European Commission does not regulate cheese coliforms limits. Coliform bacteria were detected in cheese samples in zero time, while it was undetectable in T samples after 1 week of refrigerated storage until the end of 4 weeks of analysis. One study by Salem et al.^45^ incorporated LSE into gelatin film and showed preserving effects on cheese quality. Abdel Aziz and Osheba^46^ suggested that spraying fish fillets with an aqueous extract of *L. sativum* reduced the microbial load and improved the shelf life. The edible coating of *L. sativum* mucilage was also applied to extend the shelf life of fresh-cut potato strips^47^.

**Table 3:** Microbiological analysis of cheese.

Microorganisms groups	Storage	Cheese treatments		
		C	B	T
Mesophilic bacteria	Zero time	4.4 ± 0.0^Ac^	4.4 ± 0.0^Ac^	4.4 ± 0.0^Ab^
	W1	6.9 ± 0.2^Ab^	6.6 ± 0.1^A**B**b^	6.3 ± 0.2^Ba^
	W2	7.7 ± 0.2^Ba^	8.0 ± 0.0^Aa^	6.5 ± 0.0^Ca^
	W3	ND	ND	6.6 ± 0.2^a^
	W4	ND	ND	6.5 ± 0.3^a^
Psychrotrophic bacteria	Zero time	3.5 ± 0.2^Ac^	3.5 ± 0.2^Ac^	3.5^Ab^
	W1	6.4 ± 0.1^Ab^	6.3 ± 0.2^Ab^	5.6^Ba^
	W2	7.8 ± 0.1^Aa^	7.7 ± 0.3^Aa^	5.6^Ba^
	W3	ND	ND	5.6^a^
	W4	ND	ND	6.3^a^
Yeast and mold	Zero time	2.7 ± 0.1^Ab^	2.7 ± 0.1^Ac^	2.7 ± 0.1^Ac^
	W1	5.1 ± 0.2^Aa^	4.6 ± 0.4^Ab^	4.9 ± 0.2^Ab^
	W2	6.4 ± 1.0A^Ba^	7.5 ± 0.2^Aa^	5.6 ± 0.3^ABab^
	W3	ND	ND	5.6 ± 0.5^ab^
	W4	ND	ND	6.4 ± 0.8^a^
Coliform bacteria	Zero time	2.6 ± 0.0^Ac^	2.6 ± 0.0^Ac^	2.6 ± 0.0^Ac^
	W1	2.8 ± 0.1^Ab^	2.8 ± 0.1^Ab^	N
	W2	3.0 ± 0.0^Aa^	3.0 ± 0.0^Aa^	N
	W3	ND	ND	N
	W4	ND	ND	N

(C) Uncovered cheese, (B) covered with A film, (T) covered with A1 film. Results represent the mean (n) of 3 replicates ± sd. Means within the same microorganism group showing the same uppercase letters in the same row are not significantly different (p > 0.05) and means showing the same lowercase letters in the same column are not significantly different (p > 0.05). ND: not determined. N: not detected.

### Decay evaluation

**Figure 6:**
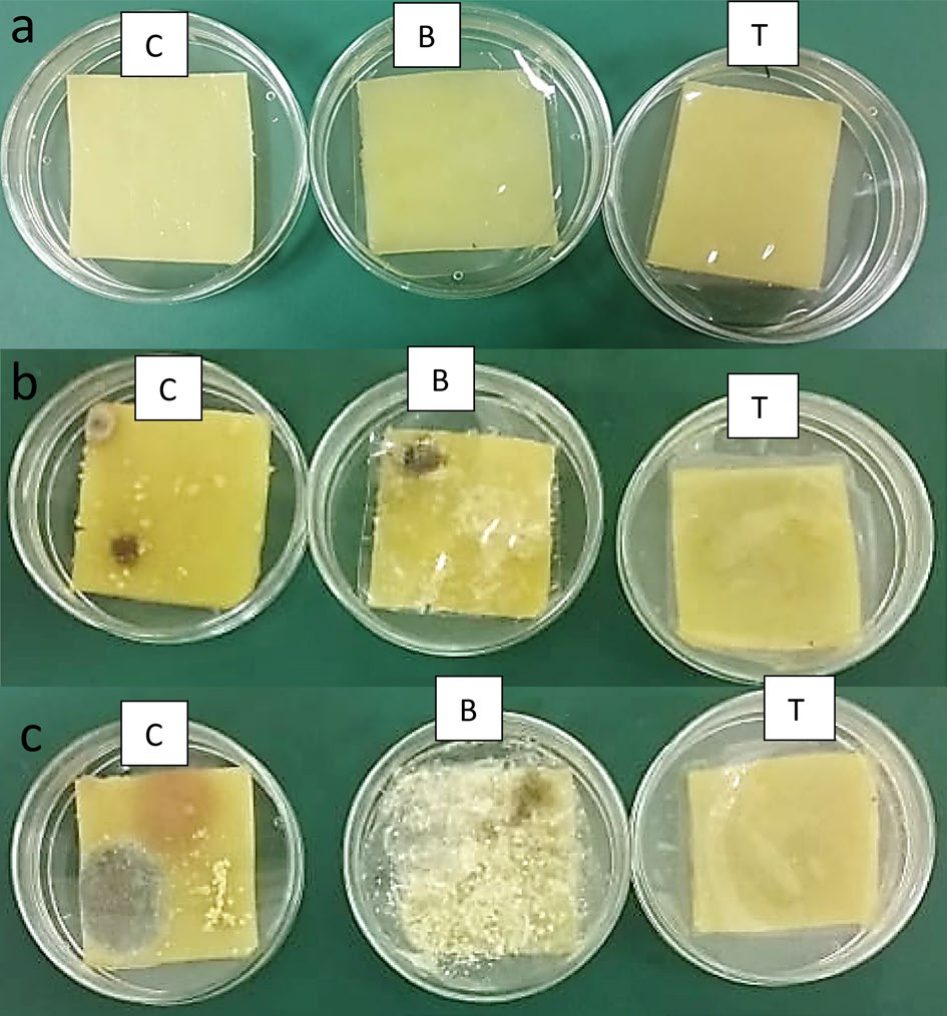
Development of visual spoilage on the cheese surface of (C) control uncovered cheese, (B) cheese covered with film (A), and (T) cheese covered with film (A1). (**a**) Zero time, (**b**) after 2 weeks, and (**c**) after 4 weeks of refrigerated storage.

**Figure 7:**
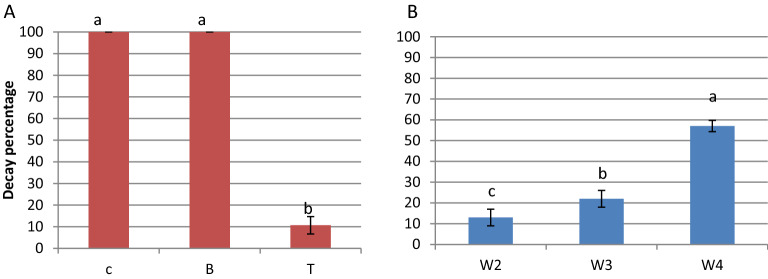
Decay percentage of different cheese treatments. (**a**) Decay (%) of different cheese samples on the second week of storage. (**b**) Decay (%) of T samples during the storage period. Data are the means ± S.E. (n = 15). Different letters indicate significant differences (p < 0.05).

Visual spoilage of cheddar cheese was followed during 4 weeks of refrigerated storage. Figure 6a–c shows that, after 2 weeks of cold storage, 100% of the control samples uncovered with film and B samples covered with PVA film had visual spoilage by yeast colonies and filamentous fungi. On the other hand, approxematly 10% of T cheese samples covered with PVA-LSE were spoiled. By the 3rd and 4th weeks, 22% and 57% of T samples showed visible yeast colonies but no filamentous fungi. Moreover, the invasion of yeast colonies on the cheese surface was less than that in the other treatments (Fig. 7).

### Fatty acid profile of lipids extracted from cheese

**Figure 8:**
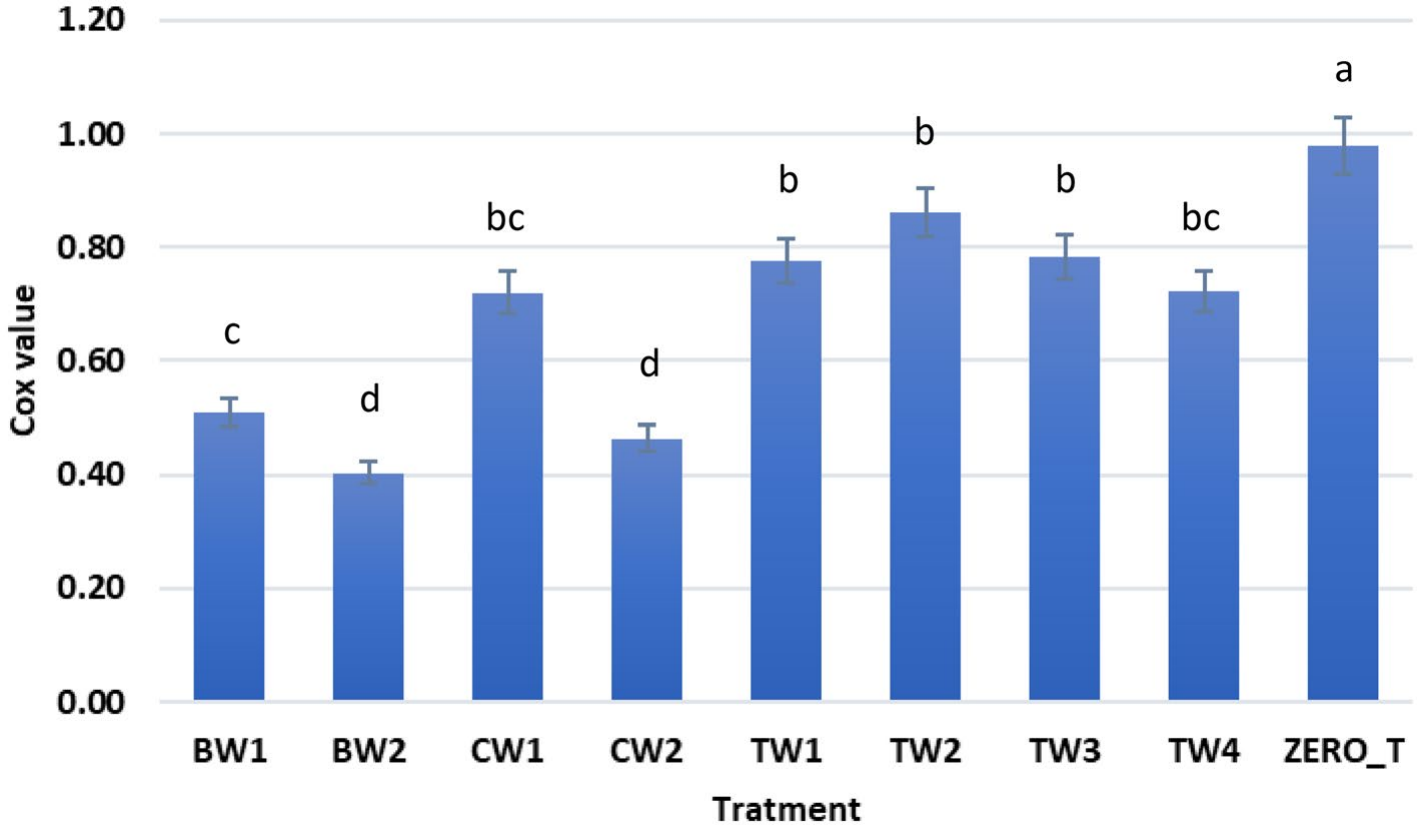
Oxidizability values of cheese samples.

In this study, SFAs predominated in all cheeses examined (63.68–74.93%). A significantly lower content of polyunsaturated FAs (0.13–4.44%) was also found in cheeses in the 4-week storage period. Additionally, all cheese samples contained the major SFAs palmitic acid, myristic acid, and stearic acid (Table 4). Moreover, the MUFA content was significantly higher (p < 0.05) in the coated film-treated samples with LSE than in the control samples (Table 4). In all treatments, oleic acid (C18.1) was the major MUFA, while linoleic acid (C18.2) and linolenic acid (C18.3) are the most important PUFAs, but they were found at lower percentages. The SCFA content, except capric and lauric acid, did not differ significantly between the controls and treatment groups. The pure film-treated cheese without LSE and the control samples contained *trans* FAs with a percentage ranging between 1.11–2.10%, while the cheese samples coated with prepared films treated with LSE did not contain *trans* fats during the different storage periods. Tsuzuki^48^ reported that adding antioxidants to fats and oils (during processing and cooking) facilitated the control of the heat-induced *trans* fat formation of unsaturated fats. This may explain why the antioxidant effect of LSE helps prevent the formation of *trans* fats in coated cheese samples. The oxidizability (Cox value) in fat extracted from different cheese samples was calculated as 0.40–0.98 (Fig. 8); the high Cox value demonstrates the oxidation resistance. Prandini et al.^48^ indicated that cheese made from cow milk contained SFA ranging from 65.23 to 68.52%, the MUFA content varied from 27.90 to 31.19%, and the PUFA content ranged from 3.48 to 4.17%^49^. Paszczyk and Łuczyńska^50^ established that the MUFA and PUFA content in commercial cheese made from cow milk was 27.92% and 3.31%, respectively.

**Table 4:** Fatty acids profile of lipids extracted from cheese samples.

Fatty acids	Area %								
	BW1	BW2	CW1	CW2	TW1	TW2	TW3	TW4	ZERO_T
Butyric acid (C4.0)	ND	ND	0.36	ND	ND	0.12	0.13	ND	ND
Caproic acid (C6.0)	0.67	1.99	2.13	1.81	2.85	2.84	1.61	1.88	2.95
Caprylic acid (C8.0)	1.69	3.57	2.55	3.45	1.56	2.64	2.45	2.32	1.52
Capric acid (C10.0)	5.01	8.49	6.15	7.6	3.82	4.95	6.54	4.98	3.47
Decenoic Acid (C10.1)	0.48	0.74	0.48	0.64	0.37	0.49	0.48	0.54	0.41
Lauric acid (C12.0)	6.48	8.57	6.99	8.01	5.12	5.98	7.69	6.21	4.89
Tridecanoic acid (C13.0)	ND	ND	0.28	ND	ND	ND	ND	ND	ND
Myristic acid (C14.0)	13.74	14.07	12.74	14.74	11.99	12.37	13.9	13.12	11.68
Myristoleic acid (14.1)	0.92	1.11	0.97	1.01	0.94	0.98	1.12	1.01	0.99
Myristolinoleic (C14.2)	0.61	ND	0.66	ND	ND	ND	ND	0.77	ND
Pentadecanoic acid (C15.0)	1.31	1.26	1.25	1.3	1.46	1.87	1.64	1.47	1.74
Palmitic acid (C16.0)	28.47	26.25	25.24	26.86	27.57	25.38	24.37	26.54	26.28
Palmitoleic acid (C16.1)	2.05	2.9	1.61	2.39	1.55	2.37	2.54	2.14	2.15
Palmitoleic acid (C16.1, n7)	0.58	ND	0.11	ND	ND	ND	ND	ND	0.07
Heptadecanoic acid (C17.0)	0.61	1.32	0.59	ND	ND	0.87	ND	0.66	ND
Cis-10-Heptadecanoic acid (C17.1)	0.40	0.62	ND	ND	0.09	ND	ND	ND	ND
Stearic acid (C18.0)	10.51	8.70	8.40	8.96	8.55	7.89	7.98	8.68	7.97
Oleic acid (C18.1n9c)	20.58	16.33	24.56	18.55	27.44	23.07	22.03	23.61	26.77
Oleic acid (C18.1n9t)	1.24	1.11	1.01	1.07	ND	ND	ND	ND	ND
Linoleic acid (C18.2n6c)	1.32	1.05	3.06	1.19	3.71	4.35	3.82	3.22	4.44
Linoleic acid (C18.2n6t)	0.86	ND	0.52	0.75	ND	ND	ND	ND	ND
α- Linolenic acid (C18.3n3)	0.78	0.61	0.74	0.72	0.55	0.84	0.78	0.72	1.18
γ- Linolenic acid (C18.3n6)	0.75	0.64	0.22	0.13	1.15	1.25	1.22	1.27	0.31
Arachidic acid (C20.0)	0.97	0.71	0.92	0.85	1.29	1.74	1.7	0.89	1.6
Behenic acid (C22.0)	ND	ND	ND	ND	ND	ND	ND	ND	1.58
**Saturated FA**	**69.46**	**74.93**	**67.6**	**73.58**	**64.21**	**66.65**	**68.01**	**66.75**	**63.68**
**Unsaturated FA**	**30.57**	**25.11**	**33.94**	**26.45**	**35.8**	**33.35**	**31.99**	**33.28**	**36.32**
***trans* fat**	**2.1**	**1.11**	**1.53**	**1.82**	**0**	**0**	**0**	**0**	**0**

*ND* Not detectable.

## Conclusion

Active packaging films for cheddar cheese were prepared from blends containg different ratios of PVA and LSE. Hyperbranched polyamide amine (PAMAM) as a plasticizer was added in small amount in all formulations. It was found that increasing the LSE amount in the film composition reduced the thermal and mechanical properties. Additionally, the antioxidant activity of the film containing highest amount of LSE was ≈86%, and a noticeable reduction of antioxidant activity was observed by decreasing amount of LSE in the film composition. The antimicrobial activity of the packaging film was investigated against different microorganisms. The results demonstrated that the order of antimicrobial activity of films was A3 > A2 > A1. The decay percentage of cheese samples covered with the treated film was 10% after one week of storage, while samples covered with untreated films had a 100% decay percentage. Investigation of the FA profile extracted from cheese samples confirmed the presence of *trans* FA in samples covered with untreated films, while in other samples, no *trans* FAs were detected. The results of this study show that packaging films containing LSE are promising active films for dairy products that can prevent bacterial attacks and extend their shelf life.

## Data Availability

All data generated or analyzed during this study are included in this article.

## References

[1] Asgher M, Qamar SA, Bilal M, Iqbal HMN (2020). Bio-based active food packaging materials: Sustainable alternative to conventional petrochemical-based packaging materials. Food Res. Int..

[2] Álvarez-Chávez CR, Edwards S, Moure-Eraso R, Geiser K (2012). Sustainability of bio-based plastics: General comparative analysis and recommendations for improvement. J. Clean. Prod..

[3] Moreira BR, Pereira-Júnior MA, Fernandes KF, Batista KA (2020). An ecofriendly edible coating using cashew gum polysaccharide and polyvinyl alcohol. Food Biosci..

[4] Karazhiyan H, Razavi SMA, Phillips GO (2009). Rheological properties of Lepidium sativum seed extract as a function of concentration, temperature and time. Food Hydrocoll..

[5] Guo Q, Cui SW, Wang Q, Christopher Young J (2008). Fractionation and physicochemical characterization of psyllium gum. Carbohydr. Polym..

[6] Jian H-L, Lin X-J, Zhang W-A (2014). Characterization of fractional precipitation behavior of galactomannan gums with ethanol and isopropanol. Food Hydrocoll..

[7] Kang J, Cui SW, Chen J (2011). New studies on gum ghatti (Anogeissus latifolia) part I. Fractionation, chemical and physical characterization of the gum. Food Hydrocoll..

[8] Razmkhah S, Razavi SMA, Mohammadifar MA (2016). Stepwise extraction of Lepidium sativum seed gum: Physicochemical characterization and functional properties. Int. J. Biol. Macromol..

[9] Walther B, Schmid A, Sieber R, Wehrmüller K (2008). Cheese in nutrition and health. Dairy Sci. Technol..

[10] MacGibbon AKH (2020). Composition and structure of bovine milk lipids. Advanced Dairy Chemistry.

[11] Lindmark Månsson H (2008). Fatty acids in bovine milk fat. Food Nutr. Res..

[12] Summer A, Formaggioni P, Franceschi P (2017). Cheese as functional food: The Example of parmigiano reggiano and grana padano. Food Technol. Biotechnol..

[13] Faour-Klingbeil D, Todd E (2019). Prevention and control of foodborne diseases in middle-east north African countries: review of national control systems. Int. J. Environ. Res. Public Health.

[14] Choi KH, Lee H, Lee S (2016). Cheese microbial risk assessments—A review. Asian-Aust. J. Anim. Sci..

[15] Kure CF, Skaar I (2019). The fungal problem in cheese industry. Curr. Opin. Food Sci..

[16] Proulx J, Sullivan G, Marostegan LF (2017). Pulsed light and antimicrobial combination treatments for surface decontamination of cheese: Favorable and antagonistic effects. J. Dairy Sci..

[17] Al-Moghazy M, El-sayed HS, Salama HH, Nada AA (2021). Edible packaging coating of encapsulated thyme essential oil in liposomal chitosan emulsions to improve the shelf life of Karish cheese. Food Biosci..

[18] Malhotra B, Keshwani A, Kharkwal H (2015). Antimicrobial food packaging: Potential and pitfalls. Front. Microbiol..

[19] Karamkhani M, Anvar SA, Ataee M (2018). The use of active edible coatings made from a combination of Lepidium sativum gum and Carvacrol to increase shelf life of farmed shrimp kept under refrigerator condition. Iran J. Aquat. Anim. Heal..

[20] Yassin MA, Gad AAM, Ghanem AF, Abdel Rehim MH (2019). Green synthesis of cellulose nanofibers using immobilized cellulase. Carbohydr. Polym..

[21] Naeem MA, Zahran HA, Hassanein MMM (2019). Evaluation of green extraction methods on the chemical and nutritional aspects of roselle seed (*Hibiscus sabdariffa* L.) oil. OCL.

[22] Nada A, Al-Moghazy M, Soliman AAF (2018). Pyrazole-based compounds in chitosan liposomal emulsion for antimicrobial cotton fabrics. Int. J. Biol. Macromol..

[23] Naghili H, Tajik H, Mardani K (2013). Validation of drop plate technique for bacterial enumeration by parametric and nonparametric tests. Vet. Res..

[24] Cao X, Huang R, Chen H (2017). Evaluation of pulsed light treatments on inactivation of Salmonella on blueberries and its impact on shelf-life and quality attributes. Int. J. Food Microbiol..

[25] Christie, W.W. The isolation of lipids from tissues. Recommended Procedures. Chloroform-methanol (2:1,v/v) extraction and “Folch” wash. In: Lipid Analysis. Pergamon, pp 39–40 (1973)

[26] Fatemi SH, Hammond EG (1980). Analysis of oleate, linoleate and linolenate hydroperoxides in oxidized ester mixtures. Lipids.

[27] Zahran HA, Tawfeuk HZ (2019). Physicochemical properties of new peanut (*Arachis **hypogaea* L.) varieties. OCL.

[28] Rafińska K, Pomastowski P, Rudnicka J (2019). Effect of solvent and extraction technique on composition and biological activity of Lepidium sativum extracts. Food Chem..

[29] Abdel-Aty AM, Salama WH, Fahmy AS, Mohamed SA (2019). Impact of germination on antioxidant capacity of garden cress: New calculation for determination of total antioxidant activity. Sci. Hortic. (Amsterdam).

[30] Kaiyrkulova A, Li J, Aisa HA (2019). Chemical constituents of lepidium sativum seeds. Chem. Nat. Compd..

[31] Tomalia DA, Naylor AM, Goddard WA (1990). Starburst dendrimers: Molecular-level control of size, shape, surface chemistry, topology, and flexibility from atoms to macroscopic matter. Angew. Chem. Int. Ed. English.

[32] Inoue K (2000). Functional dendrimers, hyperbranched and star polymers. Prog. Polym. Sci..

[33] Kumar A, Han SS (2017). PVA-based hydrogels for tissue engineering: A review. Int. J. Polym. Mater. Polym. Biomater..

[34] Abdelghany AM, Meikhail MS, Abdelraheem GEA (2018). Lepidium sativum natural seed plant extract in the structural and physical characteristics of polyvinyl alcohol. Int. J. Environ. Stud..

[35] Amer AA, Mohammed RS, Hussein Y (2022). Development of lepidium sativum extracts/PVA electrospun nanofibers as wound healing dressing. ACS Omega.

[36] Fahami A, Fathi M (2018). Fabrication and characterization of novel nanofibers from cress seed mucilage for food applications. J. Appl. Polym. Sci..

[37] Kuan HTN, Tan MY, Hassan MZ, Zuhri MYM (2022). Evaluation of physico-mechanical properties on oil extracted ground coffee waste reinforced polyethylene composite. Polymers (Basel).

[38] Gutiérrez L-F, Ratti C, Belkacemi K (2008). Effects of drying method on the extraction yields and quality of oils from quebec sea buckthorn (Hippophaë rhamnoides L.) seeds and pulp. Food Chem..

[39] Skroza D, Šimat V, Smole Možina S (2019). Interactions of resveratrol with other phenolics and activity against food-borne pathogens. Food Sci. Nutr..

[40] Abdel-Aty AM, Bassuiny RI, Barakat AZ, Mohamed SA (2019). Upgrading the phenolic content, antioxidant and antimicrobial activities of garden cress seeds using solid-state fermentation by Trichoderma reesei. J. Appl. Microbiol..

[41] Gutiérrez-Venegas G, Gómez-Mora JA, Meraz-Rodríguez MA (2019). Effect of flavonoids on antimicrobial activity of microorganisms present in dental plaque. Heliyon.

[42] Nićiforović N, Abramovič H (2014). Sinapic acid and its derivatives: Natural sources and bioactivity. Compr. Rev. Food Sci. Food Saf..

[43] Jiang Z, Wang J, Xiang D, Zhang Z (2022). Functional properties and preservative effect of P-hydroxybenzoic acid grafted chitosan films on fresh-cut jackfruit. Foods.

[44] Martin NH, Trmčić A, Hsieh T-H (2016). The evolving role of coliforms as indicators of unhygienic processing conditions in dairy foods. Front. Microbiol..

[45] Salem A, Jridi M, Abdelhedi O (2021). Development and characterization of fish gelatin-based biodegradable film enriched with Lepidium sativum extract as active packaging for cheese preservation. Heliyon.

[46] Abdel Aziz HA, Osheba ASA (2012). Microbiological quality attributes of nile perch lates niloticus fish fillets treated with aqueous extract of lepidium sativum l. Cress seeds. Egypt J. Agric. Res..

[47] Ali MR, Parmar A, Niedbała G (2021). Foods.

[48] Tsuzuki W (2011). Effects of antioxidants on heat-induced trans fatty acid formation in triolein and trilinolein. Food Chem..

[49] Prandini A, Sigolo S, Tansini G (2007). Different level of conjugated linoleic acid (CLA) in dairy products from Italy. J. Food Compos. Anal..

[50] Paszczyk B, Łuczyńska J (2020). The comparison of fatty acid composition and lipid quality indices in hard cow, sheep, and goat cheeses. Foods.

